# Impact of the updated TNM staging criteria on prediction of persistent disease in a differentiated thyroid carcinoma cohort

**DOI:** 10.20945/2359-3997000000097

**Published:** 2019-02-01

**Authors:** Carla Fernanda Nava, André B. Zanella, Rafael Selbach Scheffel, Ana Luiza Maia, Jose Miguel Dora

**Affiliations:** 1 Hospital de Clínicas de Porto Alegre Serviço de Endocrinologia Grupo de Tireoide RS Brasil Grupo de Tireoide, Serviço de Endocrinologia, Hospital de Clínicas de Porto Alegre, RS, Brasil; 2 Universidade Federal do Rio Grande do Sul Universidade Federal do Rio Grande do Sul Faculdade de Medicina Porto Alegre RS Brasil Faculdade de Medicina, Universidade Federal do Rio Grande do Sul (UFRGS), Porto Alegre, RS, Brasil

**Keywords:** Differentiated thyroid carcinoma, TNM staging, prognosis

## Abstract

**Objective:**

The 8^th^ TNM system edition (TNM-8) released in 2018 presents significant changes when compared to the 7^th^ edition (TNM-7). The aim of this study was to assess the impact of changing the TNM staging criteria on the outcomes in a Brazilian cohort of differentiated thyroid carcinoma (DTC).

**Subjects and methods:**

DTC patients, attending a tertiary, University-based hospital, were classified by TNM-7 and TNM-8. Prediction of disease outcomes status of the two systems was compared in a retrospective cohort study design.

**Results:**

Four hundred and nineteen DTC patients were evaluated, comprised by 82% (345/419) women, with mean age at diagnosis of 46.4 ± 15.6 years, 89% (372/419) papillary thyroid carcinoma, with a median tumor size of 2.3 cm (P25-P75, 1.3-3.5). One hundred and sixty patients (38%) had lymph node metastases and 47 (11%) distant metastases at diagnosis. Using the TNM-7 criteria, 236 (56%) patients were classified as Stage I, 50 (12%) as Stage II, 75 (18%) as Stage III and 58 (14%) as Stage IV. When evaluated by the TNM-8, 339 (81%) patients were classified as Stage I, 64 (15%) as Stage II, 2 (0.5%) as Stage III and 14(3%) as Stage IV. After a median follow-up of 4.4years (P25-P75 2.6-6.6), the rate of incomplete biochemical and/or structural response was 54% vs. 92% (P = 0.004) and incomplete structural response was 42% vs. 86% (P = 0.009) for patients classified as stage IV by TNM-7 vs TNM-8, respectively. Only 4 (1%) disease-related deaths were recorded.

**Conclusions:**

In our cohort, 37% of DTC patients were down staged with the application of TNM-8 (vs. TNM-7). Additionally, TNM-8 seems to better stratify the risk of structural incomplete response at follow-up.

## INTRODUCTION

Differentiated thyroid carcinoma (DTC), comprising papillary (PTC) and follicular carcinoma (FTC), accounts for the majority (> 90%) of all thyroid malignancies (1). In recent years, an increasing incidence of DTC diagnosis has been documented worldwide (2). The incremental use of imaging resources in medical practice, along with the continuous development of more sensitive techniques, led to the pandemic observation of “overdiagnosis” of DTC (2). Most of the increment in DTC diagnosis can be credited to increased detection of pre-clinical indolent tumors restricted to the thyroid gland, that present a more favorable disease profile (3,4).

The current 2015 ATA Management Guidelines for Adult Patients with DTC advise that “AJCC/UICC staging is recommended for all patients with DTC, based on its utility in predicting disease mortality, and its requirement for cancer registries” (5). In response to the change in DTC epidemiology, staging systems are being revised, to ensure a more balanced delivery of therapy for DTC, specially for those at low risk of disease-related morbidity/mortality.

The 8^th^ edition (TNM-8) of the American Joint Committee on Cancer; Tumor, Lymph Nodes, Metastasis (AJCC/TNM) system, was incorporated into the management of DTC in January of 2018 (6). The most significant changes were the age at diagnosis cut-off (from 45 to 55 years) and the redefinition of the T3b and T4a classifications. In the TNM-7, microscopic extrathyroidal extension of the tumor warranted a T3 classification. In the TNM-8, T3 is now comprised of tumors greater than 4 cm and confined to the thyroid gland (T3a) or gross extrathyroidal extension of the strap muscles (T3b). T4a is now defined as gross invasion of the subcutaneous tissue, larynx, trachea, esophagus or recurrent laryngeal nerve and T4b as gross invasion of the prevertebral fascia or major vessels (6,7).

The update in the TNM staging criteria aims to improve prediction of disease specific and overall survival, allocating patients at risk of persistent/recurrent disease for more advanced TNM stages, qualifying diagnostic work up and interventions at follow-up (8).

In Brazil we have scarce data on the clinical impact of updating the TNM criteria. Thus, the objective of this study was to assess how changing the TNM staging criteria affects the prediction of long term outcomes in a cohort of Brazilian DTC patients in tertiary, University-based hospital.

## SUBJECTS AND METHODS

### Patients and study design

The patients included were followed in a retrospective cohort of DTC patients from the Thyroid Outpatient Clinic of the Thyroid Unit, Endocrine Division of Hospital de Clínicas de Porto Alegre (HCPA), a tertiary care, university teaching hospital in southern Brazil. From 2009 to 2015, all consecutive patients with a histological diagnosis of DTC and data available to be classified by TNM-7 and TNM-8 staging systems were included. The study was approved by the ethics committee of the institution (CAAE 68434617.5.0000.5327/GPPG 17-0482).

### Treatment protocol and follow-up

Our DTC treatment protocol consists of performing total thyroidectomy and administration of radioactive iodine (RAI) remnant ablation activity according to the ATA risk assessment. Decisions regarding cervical lymph node dissection were made at the discretion of the surgical team from the center where the patients underwent the first surgery. Duration of follow-up was defined as the time between the first surgery and the last medical visit to the clinic.

Our ablation protocol used RAI activities prescribed at the attending physician's discretion. The RAI was administered in a stimulated TSH condition of endogenous hypothyroidism (TSH > 30 mUI/L), after withdrawing levothyroxine (at least 3-4 weeks without thyroid hormone). A post-treatment whole body scan (post-treatment WBS) was performed seven to ten days after RAI administration.

In the first evaluation, the following data were recorded for each patient: demographics, tumor characteristics [e.g., histological features (papillary thyroid carcinoma or follicular thyroid carcinoma – including Hurthle cell carcinoma), extension and lymph node involvement] and treatment (e.g., surgery, RAI remnant ablation, and other interventions). Each patient was classified using the 7^th^ and 8^th^ editions of the TNM/AJCC staging system (I, II, III, or IV) (6,9). N0 status was defined with postoperative neck ultrasound (US) imaging or pathological examination of patients with lymph node resection. Distant metastasis (M1) was considered present when there was a lesion outside the cervical bed on imaging (CT, MRI or scintigraphy) with: histological confirmation or post-treatment WBS uptake and/or elevated Tg. The risk of persistent/recurrent disease was assessed based on the proposed risk stratification system by the American Thyroid Association (ATA) guidelines, with patients classified into three risk groups: low, intermediate and high (5).

The follow-up protocol called for an initial assessment at 3 to 6 months after the initial treatment, which included a physical examination of the neck and measurements of the serum thyroglobulin levels under TSH suppression (Tg-T4) and anti-thyroglobulin antibody (TgAb). In a second evaluation, 6 to 12 months after the initial treatment, serum thyroglobulin (Tg) under stimulated TSH condition in endogenous hypothyroidism (TSH > 30 mIU/L) (sTg) and TgAb were measured. Neck ultrasound was performed in this first year of follow-up. At this point, the patient was classified according to disease outcome to initial therapy (see below in the outcomes section). Patients classified as having an excellent response were scheduled for annual visits, during which a physical examination of the neck and measurements of Tg-T4 and TgAb were performed. Patients with indeterminate or incomplete responses were scheduled for the same examination at least twice a year. Additional imaging studies [e.g. x-ray, bone scintigraphy, computed tomography (CT), magnetic resonance (MR)] were performed, as needed, whenever the clinical or laboratory findings raised the suspicion of persistent or recurrent disease.

### Outcomes

In the first year of follow-up, the disease outcome of initial therapy was assessed based on the serum Tg-T4 or sTg levels, neck US, and additional imaging exams, whenever indicated.

An excellent response was defined as having no clinical or imaging evidence of tumor (i.e., no imaging evidence of tumor on neck US or CT), undetectable (< 0.2 ng/mL) serum Tg-T4 levels and/or sTg levels < 1 ng/mL. Indeterminate response was defined as nonspecific findings on imaging studies, Tg-T4 detectable but < 1 ng/mL, sTg detectable but < 10 ng/mL or TgAb stable or declining in the absence of structural or functional disease. Biochemical incomplete response was defined as negative imaging and Tg-T4 ≥ 1 ng/ml or sTg levels ≥ 10 ng/mL or rising TgAb levels. Structural incomplete response was defined as structural or functional evidence of disease at any Tg-T4 level with or without TgAb. Patients who were diagnosed with persistent disease were evaluated for additional treatment (e.g., surgery, RAI, external-beam radiation or multi-kinase inhibitors), depending on the site involved and disease progression.

All patients with biochemical incomplete and structural incomplete responses were considered to have persistent disease. Recurrence was defined as new biochemical or structural evidence of the disease detected in a patient who had been previously classified as having an excellent response. Disease specific survival (DSS) was considered the time from initial surgery to last follow-up or death related to DTC.

### Laboratory analysis

Serum Tg measurements were conducted using immunoradiometric assays (from 2000 to 2002 radioimmunoassay; 2002 to 2010 electrochemiluminescence and 2010 until the present chemiluminescence) that indicated functional sensitivities of at least 0.2 ng/mL. Antithyroglobulin antibodies were measured using the passive agglutination method from 2000 to 2010 and by chemiluminescence from 2010 until the present. After each new technique had been implemented, the necessary procedures for standardization and validation were performed. TSH levels were measured by chemiluminescence assay from 2000 to 2006 (Immulite 2000 SIEMENS), electrochemiluminescence from 2006 to 2010 (Modular E Roche), chemiluminescence assay from 2010 to 2014 (Centaur XP Siemens) and electrochemiluminescence from 2014 until the present (Cobas E602 Roche). These tests were all conducted in the central laboratory of the HCPA.

### Statistical analysis

The clinical and laboratory data are reported as the mean ± standard deviation (SD) values or as the median and percentiles 25 and 75 (P25-75) for continuous variables, or as absolute numbers and percentages for categorical variables. Comparative analyses of frequencies were performed using Pearson Chi-Square or Fisher's Exact Test, as appropriate. Agreement for qualitative (categorical) variables was accessed using the *Cohen's Kappa* coefficient.

All tests were two-tailed, and all analyses were performed using the Statistical Package for Social Science Professional software version 20.0 (IBM Corp., Armonk, NY). A two-tailed P < 0.05 was considered statistically significant.

## RESULTS

### Patients

Four hundred and nineteen DTC patients were evaluated. Clinical and oncological characteristics of these patients are described in Table 1. The mean age at diagnosis was 46.4 ± 15.6 years, 345 (82%) were women and PTC was diagnosed in 372 (89%) patients. The median tumor size was 2.3 cm (P25-75 1.3-3.5 cm). One hundred and sixty patients (38%) had lymph node metastases, and 47 (11%) patients had distant metastases. All patients underwent total thyroidectomy ± lymph node dissection and received RAI remnant ablation. The mean RAI activity was 112.3 ± 38.1 mCi.

**Table 1:** Characteristics of 419 patients with differentiated thyroid carcinoma

Age at diagnosis (years)		46.4 ± 15.6
Female sex – n (%)		345 (82.3)
Histology – n (%)		
	Papillary	372 (88.8)
	Follicular	74 (11.2)
Tumor size (cm)		2.3 (1.3-3.5)
Lymph node metastasis (N1) – n (%)		160 (38.2)
Distant metastasis – n (%)		47 (11.2)
RAI therapy – n (%)		419 (100)
RAI activity (mCi)		112.3 ± 38.1
Follow-up (years)		4.4 (2.6-6.6)

Data are expressed as the mean ± SD, median (percentiles 25-75) or frequencies.

RAI: radioactive iodine.

### TNM-7 *vs*. TNM-8: staging comparison

Using the TNM-7 criteria, 236 (56%) patients were classified as stage I, 50 (12%) as stage II, 75 (18%) as stage III and 58 (14%) as stage IV. When evaluated by the TNM-8, 339 (81%) patients were classified as stage I, 64 (15%) as stage II, 2 (0.5%) as stage III and 14 (3%) as stage IV (Table 2).

**Table 2:** Modifications of differentiated thyroid carcinoma staging from the TNM-7 to the TNM-8 system

DTC Staging		TNM-8			
		I (n = 339)	II (n = 64)	III (n = 2)	IV (n = 14)
**TNM-7**	I (n = 236)	236 (100%)	0 (0%)	0 (0%)	0 (0%)
	II (n = 50)	36 (72%)	14 (28%)	0 (0%)	0 (0%)
	III (n = 75)	49 (65%)	26 (35%)	0 (0%)	0 (0%)
	IV (n = 58)	19 (33%)	24 (41%)	2 (3%)	14 (24%)

DTC: differentiated thyroid carcinoma.

Chi-square of p ≤ 0.0001 for TNM-7 vs. TNM-8.

The distribution of stage frequencies from TNM-7 to TNM-8 were statistically significant (p ≤ 0.0001), and distributed as follows: 236 (100%) patients who were stage I remained the same; of the 50 previously in stage II, 36 (72%) were reclassified to stage I and only 14 (28%) maintained the classification. Of the 75 that were stage III, 49 (65%) were reclassified to stage I, 26 (35%) to stage II and none remained in this classification; of the 58 patients in stage IV, 19 (33%) were reclassified to stage I, 24 (41%) to stage II, 2 (3%) to stage III, and only 14 (24%) remained in stage IV. One hundred and fifty-five (37%) patients with DTC were downstaged with the application of TNM-8 (vs TNM-7), 87 (56%) being due to the change in the age at diagnosis cut-off from 45 to 55 years and 68 (44%) to change in T3 definition (Table 3).

**Table 3:** Comparison of Tumor classification of TNM-7 and TNM-8 systems

	TNM-8								
		T1a	T1b	T2	T3a	T3b	T4a	T4b	Total
**TNM-7**	T1	**69**	**85**	–	–	–	–	–	**154**
	T2	–	–	**111**	–	–	–	–	**111**
	T3	6	24	37	**63**	**2**	–	–	**132**
	T4a	–	–	–	–	–	**13**	–	**13**
	T4b	–	–	–	–	–	–	**4**	**4**
	**Total**	**75**	**109**	**148**	**63**	**2**	**13**	**4**	**414**

### TNM-7 vs. TNM-8: ATA Risk

Using TNM-7, 163/233 (70%) stage I patients were classified as low risk, 63/233 (27%) as intermediate risk and 7/233 (3%) as high risk. Stage II patients were classified as follows: 31/50 (62%) as low risk, 6/50 (12%) as intermediate risk and 13/50 (26%) as high risk. Of those in stage III, 30/75 (40%) were classified as low risk, 44/75 (59%) as intermediate risk and 1/75 (1%) as high risk. Of those in stage IV, 8/58 (14%) were stratified as low risk, 23/58 (40%) as intermediate risk and 27/58 (47%) as high risk.

Applying the TNM-8, stage I patients were stratified as low risk in 212/335 (63%) cases, as intermediate risk in 113/335 (34%) and as high risk in 10/335 (3%). Those in stage II were classified as low risk in 20/65 (31%), as intermediate risk in 23/65 (35%) and as high risk in 22/65 (34%). All stage III (n = 2) and stage IV (n = 14) patients were classified as high risk (100%) (Table 4). The greater number of reclassifications in ATA low and intermediate risk patients led to a weak level of agreement between TNM-7 and TNM-8 (*kappa* 0.14 and 0.13, respectively; p ≤ 0.0001 for both comparisons). In ATA high-risk patients, the agreement between the two classifications was considered moderate (*kappa* 0.54, p ≤ 0.0001).

**Table 4:** ATA risk according to differentiated thyroid carcinoma staging using the TNM-7 and TNM-8 systems

ATA risk	TNM-7 Stage n (%)				TNM-8 Stage n (%)			
	I	II	III	IV	I	II	III	IV
Low	163 (70)	31 (62)	30 (40)	8 (14)	212 (63)	20 (31)	0 (0)	0 (0)
Intermediate	63 (27)	6 (12)	44 (59)	23 (40)	113 (34)	23 (35)	0 (0)	0 (0)
High	7 (3)	13 (26)	1 (1)	27 (47)	10 (3)	22 (34)	2 (100)	14 (100)

*Kappa* for TNM-7 vs. TNM-8 according to ATA risk: 0.14 for low risk (p ≤ 0.0001); 0.13 for intermediate risk (p ≤ 0.0001) and 0.54 for high risk (p ≤ 0.0001).

### TNM-7 vs. TNM-8: disease outcomes

Regarding disease outcomes after initial therapy, we verified an excellent response status in 47/223 (21%), 9/49 (18%), 21/71 (30%) and 6/58 (10%) for patients at stage I, II, III and IV of TNM-7, respectively. While using the TNM-8 at the same point in time, an excellent response status was as follows: 76/322 (24%), 7/63 (11%), 0/2 (0%) and 0/14 (0%) for patients at stage I, II, III and IV, respectively. Indeterminate status was observed in 99/223 (44%), 10/49 (20%), 29/71 (41%) and 18/58 (31%) for patients at stage I, II, III and IV of TNM-7, respectively; and using the TNM-8 we found 135/322 (42%), 19/63 (30%), 1/2 (50%) and 1/14 (7%) for patients at stage I, II, III and IV, respectively. Biochemical incomplete response was the disease status in 40/223 (18%), 13/49 (27%), 11/71 (15%) and 7/58 (12%) for patients at stage I, II, III and IV of TNM-7, respectively; and while using TNM-8 62/322 (19%), 8/63 (13%), 1/2 (50%) and 0/14 (0%) for patients at stage I, II, III and IV, respectively. Structural incomplete response frequencies were 37/223 (17%), 17/49 (35%), 10/71 (14%) and 27/58 (47%) for patients at stage I, II, III and IV using TNM-7, respectively; and in 49/322 (15%), 29/63 (46%), 0/2 (0%) and 13/14 (93%) for patients at stage I, II, III and IV using TNM-8 (Table 5).

**Table 5:** Disease outcomes after initial treatment according to differentiated thyroid carcinoma staging using the TNM-7 and TNM-8 systems

Treatment response	TNM-7 Stage n (%)				TNM-8 Stage n (%)			
	I	II	III	IV	I	II	III	IV
Structural incomplete	37 17)	17(35)	10 (14)	27 (47)	49 (15)	29 (46)	0 (0)	13 (93)
Biochemical incomplete	40 (18)	13 (27)	11 (15)	7 (12)	62 (19)	8 (13)	1 (50)	0 (0)
Indeterminate	99 (44)	10 (20)	29 (41)	18 (31)	135 (42)	19 (30)	1 (50)	1 (7)
Excellent	47 (21)	9 (18)	21 (30)	6 (10)	76 (24)	7 (11)	0 (0)	0 (0)

*Kappa* for TNM-7 vs. TNM-8 according to initial treatment response: 0.13 for excellent response (p = 0.006); 0.19 for indeterminate response (p ≤ 0.0001); 0.07 for biochemical incomplete response (p = 0.26) and 0.56 for structural incomplete response (p ≤ 0.0001).

**Figure 1:**
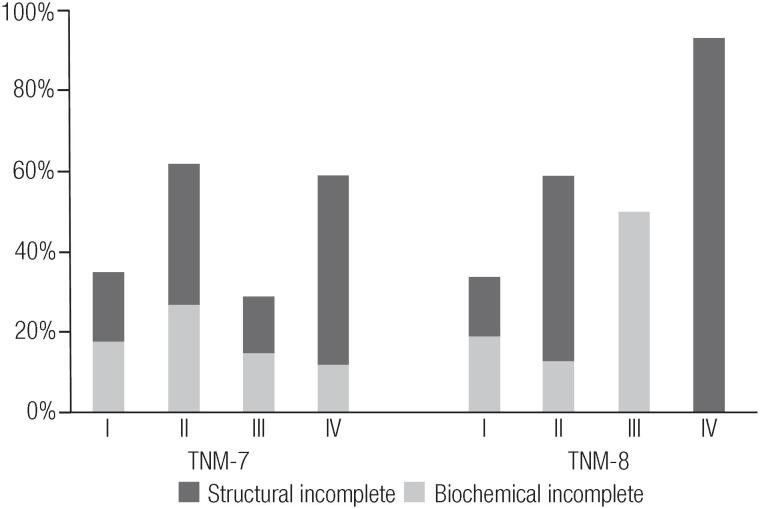
Differentiated thyroid carcinoma biochemical incomplete and structural incomplete responses to therapy at long term follow-up, according to TNM-7 and TNM-8.

After a median follow-up of 4.4 years (P25-P75 2.6-6.6), the majority of the patients maintained the same response to therapy status or were downgraded to a more favorable status, a finding observed with both TNM staging systems (p ≤ 0.001). The rate of excellent response in stage I patients was 39% vs. 38% for TNM-7 and TNM-8; in stage II patients it was 26% vs. 17%, in stage III patients it was 36% vs 0% and in stage IV patients 14% vs. 0%. The rate of indeterminate responses was 40% vs 41% in stage I patients; 36% vs. 34% in stage II patients; 45% vs. 50% in stage III patients and 32% vs. 7% in stage IV patients. The rate of biochemical incomplete response was 13% vs. 12% in stage I patients; 14% vs. 6% in stage II patients; 3% vs. 50% in stage III patients and 12% vs. 7% in stage IV patients. The rate of structural incomplete response was 8% vs. 8% in stage I patients, 24% vs. 42% in stage II patients; 16% vs. 0% in stage III patients and 42% vs. 86% (p = 0.009) in stage IV patients for TNM-7 and TNM-8, respectively (Table 6, Figure 1). The agreement level was considered moderate only for patients with incomplete structural response (*kappa* 0.51, p ≤ 0.0001).

**Table 6:** Disease outcomes at long-term follow-up according to differentiated thyroid carcinoma staging using the TNM-7 and TNM-8 systems

Treatment response	TNM-7 Stage n (%)				TNM-8 Stage n (%)			
	I	II	III	IV	I	II	III	IV
Structural incomplete	18 (8)	12 (24)	12 (16)	24 (42)	27 (8)	27 (42)	0 (0)	12 (86)
Biochemical incomplete	29 (13)	7 (14)	2 (3)	7 (12)	39 (12)	4 (6)	1 (50)	1 (7)
Indeterminate	87 (40)	18 (36)	34 (45)	18 (32)	133 (41)	22 (34)	1 (50)	1 (7)
Excellent	86 (39)	13 (26)	27 (36)	8 (14)	123 (38)	11 (17)	0 (0)	0(0)

*Kappa* for TNM-7 vs. TNM-8 according to treatment response at long-term follow-up: 0.13 for excellent response (p ≤ 0.001); 0.13 for indeterminate response (p ≤ 0.001); 0.26 for biochemical incomplete response (p = 0.002) and 0.51 for structural incomplete response (p ≤ 0.0001).

Recurrence was observed only on 3/419 (0.7%) patients during follow-up period with a median time of 2.6 years.

It was not possible to access the impact of the reclassification on DSS due to the low mortality rate observed in our cohort (n = 4, 1%). The four deaths occurred in TNM-7 stage IV patients (n = 4, 100%), and in TNM-8 stage II (n = 2, 50%) and stage IV (n = 2, 50%) patients.

## DISCUSSION

The classification of DTC patients using staging systems is a crucial step in the care of these patients, since it allowed the healthcare team to determine the best therapeutic and follow-up approach. This study examined the impact of updating the TNM staging system from TNM-7 to TNM-8 on the reclassification of DTC patients in a Brazilian cohort, showing that the TNM-8 reclassified 37% of patients to lower stages. We also compared the prognostic information from both systems, and observed that TNM-8 was superior in predicting disease status at follow-up.

In recent years the incidence of DTC has been increasing with a more frequent diagnosis of low-risk and indolent tumors (2). These patients do not benefit from an aggressive approach, but may be exposed to the risks of overtreatment. The impact of updating the TNM-7 staging system to TNM-8 has been explored by some studies (8,10,11). In our study the majority of reclassifications occurred due to age at the diagnosis cut-off change (n = 87, 56%). The change in age at the diagnosis cut-off was proposed and validated by Nixon and cols. in a multicenter study of 9,500 patients (12,13). Older patients with more advanced disease tend to have a worse prognosis and this is usually proportional to age at diagnosis. Nixon and cols. showed that increasing the age cut-off point from 45 to 55 years resulted in a better prediction of outcomes, and prevented low risk patients from being overstaged and, consequently, overtreated. The results of Kim and cols. (14) corroborate these findings, and showed that a cut-off of 55 years was better at predicting DSS. In our population age cut-off numbers were in accordance with other reports, and seem to better stratify low and high risk patients. Twenty percent of all patients in our study were downstaged because of the change in cut-off for age at diagnosis, which is in line with data from Kim and cols. (10) that found 27%, and Pontius and cols. results of 26% (SEER cohort) and 26% (NCDB cohort) (11).

Using TNM-8, we observed that stage I patients are mostly classified as ATA low risk group, stage II patients are evenly distributed between low, intermediate, and high risk and patients in stages III and IV are exclusively at ATA high risk. When we compared these figures with the classification obtained using TNM-7, we noticed that patients in stage I were also mostly in the low-risk group, while patients in stage II were distributed between low and high risk, those in stage III between low and intermediate risk and those in stage IV distributed in all three groups. Taken together these findings suggest that the TNM-8 serves as a good predictor for relapses and complications related to the disease.

Regarding the prediction of disease outcomes of the TNM-8, initially and after a median of 4.4 years (P25-P75 2.6-6.6) follow-up, we observed that patients with an excellent response were only those initially classified as stage I and II, those with indeterminate response were mainly patients in stage I and II, also including some stage III and IV. The groups of patients with biochemical incomplete and structural incomplete responses were comprised by patients with TNM-8 stage I to IV. It is worth mentioning that 86 to 93% of TNM-8 stage IV patients were on incomplete structural response and none of them had an excellent response. While comparing the two staging systems, TNM-8 classifies patients with a more severe disease spectrum as stages III and IV, identifying groups that benefit from a more aggressive treatment and follow-up approaches.

Our study has some limitations. The fact that only two patients remained in stage III after reclassification to TNM-8, makes it difficult to evaluate disease outcome prediction for this subgroup. Also, all patients included in this study underwent total thyroidectomy and received radioiodine, which limits the extrapolation of our results to patients submitted to partial thyroidectomy and to those who did not receive radioiodine. Notwithstanding, one should note that our data reflect a real clinical practice scenario at a tertiary care center, where all patients have access to standardized treatment protocols. Additionally, our data comprise a considerable number of patients, and it is one of the first studies providing Brazilian information on the subject.

In summary, 37% of DTC patients were downstaged with the application of TNM-8 (vs. TNM-7). The TNM-8 incorporates a large number of patients to lower stages, retaining the favorable prediction performance for stages I and II. It also selects more advanced DTC disease to stages III and IV. These findings confirm that the application of TNM-8 to our population is valid, and superior to TNM-7, regarding prediction of poor DTC outcomes. The use of TNM-8 has the potential to benefit a large number of patients, helping deliver the appropriate intensity of care according to disease prognosis.
